# 12-month prevalence of self-reported medical diagnoses of depression in Germany

**DOI:** 10.17886/RKI-GBE-2017-069

**Published:** 2017-10-09

**Authors:** Julia Thom, Ronny Kuhnert, Sabine Born, Ulfert Hapke

**Affiliations:** Robert Koch Institute, Department of Epidemiology and Health Monitoring, Berlin

**Keywords:** DEPRESSION, MEDICAL DIAGNOSIS, ADULTS, HEALTH MONITORING, GERMANY

## Abstract

Depression is a frequent mental disorder and has a growing importance in health care provision. In GEDA 2014/2015-EHIS, 9.7% of women and 6.3% of men self-reported having received a medical diagnosis of depression during the past 12 months. For both genders, the rate of self-reported diagnoses of depression is highest in the 45- to 64-year age group. Education thereby plays a significant role. Prevalence for women from low education is about double that of women from high education backgrounds (12.2% compared with 6.5%). The education gradient for men is smaller (7.5% compared with 5.1%). Prevalence rates also differ sharply between federal states (for women, between 5.4% and 13.4%; for men, between 3.3% and 9.4%). These results are discussed in the light of data currently available.

## Introduction

Depression is a mental disorder that is characterised by despondency, lack of motivation, severe weariness and the loss of interest in activities that used to produce pleasure [1]. Further symptoms include difficulties concentrating, a lack of self-confidence and suicidal tendencies in more severe cases. For those affected, depression implies severe impacts on quality of life and the ability to lead a productive life [2]. Among all chronic diseases, depression accounts for the greatest number of disability-adjusted years of life [3] and is considered to be a factor in at least half of all accomplished suicides [4]. Social insurance policies document the increasing care relevance of depression and its role in cases where people become incapable of working, require rehabilitation services and/or retire [5-8]. However, based on the epidemiological data available, the rate of depression in the population is a controversial issue [9-11]. To measure the prevalence of depression, beside further indicators, health monitoring at the Robert Koch Institute also collects data on self-reported medical diagnoses of depression.

## Indicator

To survey self-reported medical diagnoses of depression, the GEDA 2014/2015-EHIS survey used self-administered paper-based and online questionnaires. Respondents were asked, ‘During the past 12 months, have you had one of the following diseases or disorders?’, followed by a list of diseases which also included depression. In the face of previous surveys and to increase the interpretive and comparative value of this data, the discussion in the following sections only considers respondents who said they had suffered from depression during the past 12 months and also reported having been ‘diagnosed at least once by a doctor’ with depression. This led to the exclusion of 26.4% (n=657) of respondents who reported depression during the past 12 months but failed to provide a lifetime medical diagnosis.

**GEDA 2014/2015-EHIS**

**Data holder:** Robert Koch Institute

**Aims:** To provide reliable information about the population’s health status, health-related behaviour and health care in Germany, with the possibility of a European comparison

**Method:** Questionnaires completed on paper or online

**Population:** People aged 18 years and above with permanent residency in Germany

**Sampling:** Registry office sample; randomly selected individuals from 301 communities in Germany were invited to participate

**Participants:** 24,016 people (13,144 women; 10,872 men)

**Response rate:** 26.9%

**Study period:** November 2014 - July 2015

**Data protection:** This study was undertaken in strict accordance with the data protection regulations set out in the German Federal Data Protection Act and was approved by the German Federal Commissioner for Data Protection and Freedom of Information. Participation in the study was voluntary. The participants were fully informed about the study’s aims and content, and about data protection. All participants provided written informed consent.

More information in German is available at

www.geda-studie.de

Whilst such an approach allows for efficient estimates on the prevalence of depression and is also widely used in international health surveys [12, 13], the approach is nonetheless tied to numerous prerequisites and therefore also has its limitations. Respondents need to have 1) consulted a physician; 2) received the diagnosis of depression; 3) this diagnosis needs to meet the diagnostic criteria; and 4) be reported by a physician. When taking part in the survey, the respondent moreover needs to 5) remember having received the diagnosis and 6) be willing to report the diagnosis. Furthermore, this is based on the assumption that psychological psychotherapists who offer specialist medical care and also provide diagnoses of depression are categorised as a sub-group within the larger group of physicians.

The analyses are based on data from 23,179 participants aged 18 years and older (12,777 women and 10,402 men) with valid data on self-reported medical diagnoses of depression. The calculations were carried out using a weighting factor that corrects for deviations within the sample from the German population (as of 31 December 2014) with regard to gender, age, district type and education. The district type reflects the degree of urbanisation and accounts for the regional distribution in Germany. The International Standard Classification of Education (ISCED) was used to classify the responses provided on educational level [14]. Differences between these groups are interpreted as statistically significant if the respective confidence intervals do not overlap.

A detailed description of the methodology used in the GEDA 2014/2015-EHIS study can be found in Lange et al. 2017 [15] as well as in the article German Health Update: New data for Germany and Europe, which was published in Issue 1/2017 of the Journal of Health Monitoring.

## Results and discussion

This section presents the results of the analyses, discusses them in the context of further findings from health monitoring and contrasts them with an analysis of the data received from health insurance funds.

The 12-month prevalence of self-reported medical diagnoses of depression in the overall population was 8.1% (Table 1). Women (9.7%) report the diagnosis of depression significantly more often than men (6.3%). Prevalence in both genders is highest in the 45- to 64-year group. These findings confirm the known gender imbalance for mental disorders. The higher prevalence of depression among women compared with men is a classic and apparently stable epidemiological finding, a fact which is confirmed by studies that used numerous different forms of measurement, were implemented in various countries and over long periods of time [16]. Differences between the genders also exist regarding their willingness to seek help because faced with a depressive disorder women are more likely to seek therapy than men [17]. The debate on differences between the genders explains these facts by pointing to both biological mechanisms and the effects of gender roles as well as factors of social stress. On the other hand, these differences are also interpreted as a distortion which results from a selection of diagnostic criteria that more typically reflects female symptoms of depression and therefore underrates depression among men [18, 19].

Increasing levels of education almost halve the prevalence of self-reported medical diagnoses of depression in the overall population (low education of 10.5% vs. high education background of 5.6%, data not shown). The education gradient in the group of women up to the age of 64 with a diagnosed depression is stronger and statistically more relevant than for men. Besides age and gender, the year of data collection also impacts the correlation between education and the prevalence of self-reported medical diagnoses of depression [20-22]. When income and professional status are considered as factors next to education, this leads to equally inconsistent patterns [23-25].

**Figure 1:**
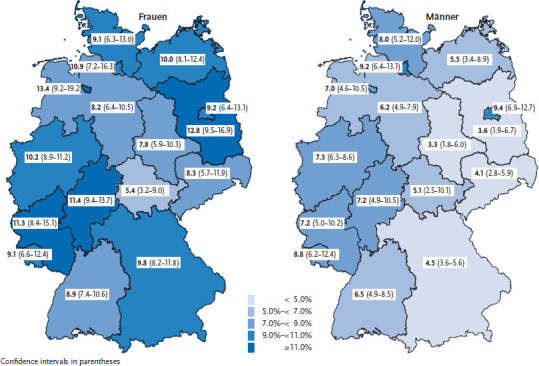
12-month prevalence of self-reported medical diagnoses of depression according to gender and federal state (n=12,777 women; n=10,402 men) Source: GEDA 2014/2015-EHIS

Prevalence rates of self-reported medical diagnoses of depression vary considerably between federal states. Prevalence is highest in the city states (13.4% of women in Bremen and 9.4% of men in Berlin) (Figure 1). Prevalence in the federal states that report the lowest rates of self-reported medical diagnoses of depression is less than half of this and affects 5.4% of women in Thuringia and 3.3% of men in Saxony-Anhalt. Excluding Bavaria, where prevalence is low, the map reveals an east to west gradient for men. Surveys from previous years [25] and data from health insurance funds [6, 11] evidence comparable differences between federal states. When comparing urban and rural areas, both of these sources of data highlight that prevalence is highest in the major cities and lowest in provincial towns [11, 23, 24]. In the accounts data from statutory health insurance funds, the frequency of depression diagnoses at the level of individual districts can vary by the factor 3 (between 5.3% and 18.2%) [26] and non-associated towns (between 7.2% and 21.4%) [11] even if regional differences are adjusted for age, gender and physical morbidity. Complex differences between regions must be taken into account to explain the unequal spatial distribution, such as varying concentrations of risk and protective factors, local factors that influence how willing the population is to seek help, local availability of treatment options as well as the frequency with which depression being treated is recognised and documented [26, 27]. As evidenced by a comparison with surveys from the past few years, the number of self-reported medical diagnoses of depression is no longer rising. Whereas in GEDA 2009, 8% of women and 4.5% of men reported depression [20], GEDA 2014/2015-EHIS results are comparable to the findings presented in GEDA 2012 (women 9.8%; men 6.1%) [22]. However, it has to be considered that the form of data collection has changed between these older surveys (a telephone interview) and GEDA 2014/15-EHIS (a self-administered paper-based or online questionnaire), which might have influenced responses.

This trend is also reflected in the diagnoses of depression according to the accounting data of health insurance funds. This data reveals a continuous increase in the reporting of medical classifications by physicians related to depressive disorders as a cause of incapacity to work over the past few years [6-8]. An evaluation of Company Health Insurance Fund (BKK) data reveals that depression-related absences from work more than doubled between 2003 and 2013 [8]. Using health insurance fund data to calculate values for the 12-month prevalence of depression would lead to rates between 10% and 13% depending on individual funds [11, 26, 28, 29]. Differences between the self-reported medical diagnoses of depression published in GEDA 2014/2015-EHIS and depression diagnoses as recorded by insurance funds exist for example concerning age distribution [6, 20-23, 26, 28]. These facts indicate conceptual differences between the data collected in surveys and accounting data [30]. Accounting data for example depends highly on the capacity of doctors to provide correct medical classifications and the validity of this data on depressive disorders is questionable [29, 31]. On the other hand, the significance of the survey data presented here depends on the degree with which the survey represents the population (response bias) as well as the above-mentioned limitations regarding the indicator itself (willingness to seek help, recall and reporting bias).

Whether a self-reported medical diagnosis of depression actually indicates depression according to clinical diagnostic criteria was a question that was analysed using data from the German Health Interview and Examination Survey for Adults (DEGS1) and its additional mental health module (DEGS1-MH) [32]. Standardised clinical interviews according to current classification criteria detect depression in only 37.2% of respondents who self-reported a medical diagnosis of depression during the past 12 months. 36.2% fulfil the criteria for a different mental disorder, whereas in 26.6% of cases the diagnosis reveals no mental disorder. On the other hand, only 33.0% of those who are diagnosed with depression in a clinical interview report a medical diagnosis of depression. Estimates on the prevalence of depression therefore both under- and overestimate the number of cases depending on whether diagnostic criteria or self-reported medical diagnoses are used as a basis. Surveys that collect epidemiological data and link it to the data from health insurance funds could provide more differentiated results (data linkage).

If, instead of looking at the diagnoses physicians provide, we use a questionnaire to survey the presence of individual symptoms of depression during the past two weeks (PHQ-8 [33, 34]), 10.1% of the population show depressive symptoms. Women are affected more often than men, there are clear regional differences and contradictory findings related to age. Whereas women aged 18 to 29 show the highest rates of depressive symptoms, it is women aged 45 to 64 who most often report a medical diagnosis of depression. For men, the number of those with depressive symptoms nearly halves at age 65 and above, but remains constant up to that age. This also highlights the fact that pressures can only be reflected in documented diagnoses once patients turn to a doctor. Compared to men, women seek medical consultation more often, a fact which is also true for younger people compared to older people [35]. Physicians who diagnose depressive symptoms in a patient do not necessarily medically classify these as depression. This is the case when for example the number and severity of depressive symptoms is low and do not therefore fulfil the general diagnosis of the disorder or when patients present further symptoms that are then collectively classified as a different mental disorder.

The data on self-reported medical diagnoses of depression published in the health survey enable, as long as we consider the limitations described, a description of the people who receive the diagnosis of depression in the healthcare system. The socio-demographic, socio-economic and regional imbalances in rates of diagnosis of depression reflect as many differences in morbidity as levels of care provision between different groups in the population. An analysis of data based on standardised diagnosed depression within a clinical interview compared to self-reported medical diagnoses highlights that different indicators in epidemiology and healthcare services diagnose reveal different groups of people with depression. Clarifying these discrepancies could contribute to a provision of services according to need, for example through an increased use of screening instruments in medical practice.

## Key statements

- 9.7% of women and 6.3% of men reported a medical diagnosis of depression during the past 12 months.
- Significant differences in rates of self-reported medical diagnoses of depression exist between the federal states.
- Prevalence of self-reported medical diagnoses of depression is highest at age 45 to 64.
- The 12-month prevalence of self-reported medically diagnosed depression decreases with increasing education.

## Figures and Tables

**Table 1:** 12-month prevalence of self-reported medical diagnoses of depression diagnosed by a physician according to gender, age and educational level (n=12,777 women; n=10,402 men) Source: GEDA 2014/2015-EHIS

Women	%	(95% CI)	Men	%	(95% CI)
**Women total**	**9.7**	**(9.0-10.3)**	**Men total**	**6.3**	**(5.8-6.9)**
**18-29 Years**	8.1	(6.7-9.7)	**18-29 Years**	4.3	(3.2-5.9)
Low education	12.3	(8.8-16.9)	Low education	7.0	(4.2-11.2)
Medium education	7.5	(6.0-9.4)	Medium education	3.4	(2.3-4.9)
High education	3.6	(2.2-5.8)	High education	3.8	(1.5-8.9)
**30-44 Years**	9.3	(8.0-10.8)	**30-44 Years**	5.7	(4.5-7.2)
Low education	13.4	(9.3-18.9)	Low education	8.1	(4.5-14.1)
Medium education	10.2	(8.5-12.1)	Medium education	6.6	(5.2-8.4)
High education	4.8	(3.6-6.4)	High education	3.2	(2.1-5.0)
**45-64 Years**	11.8	(10.8-12.9)	**45-64 Years**	8.5	(7.5-9.6)
Low education	15.1	(12.1-18.7)	Low education	9.1	(6.5-12.6)
Medium education	11.7	(10.4-13.1)	Medium education	9.3	(7.8-11.0)
High education	9.3	(7.7-11.2)	High education	7.0	(5.7-8.5)
**≥ 65 Years**	8.0	(6.7-9.5)	**≥ 65 Years**	5.0	(4.0-6.1)
Low education	10.1	(7.9-12.8)	Low education	5.6	(3.7-8.3)
Medium education	6.9	(5.3-8.9)	Medium education	5.2	(3.8-7.1)
High education	5.3	(3.4-8.1)	High education	4.2	(2.9-5.9)
**Total (women and men)**	**8.1**	**(7.6-8.5)**	**Total (women and men)**	**8.1**	**(7.6-8.5)**

CI=Confidence interval
